# Lactate regulates the YTHDF2-FTH1 axis to promote cardiomyocyte ferroptosis and aggravate myocardial ischemia-reperfusion injury

**DOI:** 10.1038/s41598-026-35130-3

**Published:** 2026-01-08

**Authors:** Zhonghao Xiang, Bitao Xiang, Tianyu Ouyang, Yadong Long, Chengliang Zhang

**Affiliations:** 1 Department of Cardiovascular Surgery, Xiangya Hospital, Central South University, 87 Xiangya Road, Changsha, 410008 Hunan People’s Republic of China; 2 National Clinical Research Center for Geriatric Disorders, Xiangya Hospital, Central South University, Changsha, People’s Republic of China

**Keywords:** Myocardial ischemia-reperfusion injury, Lactylation, Ferroptosis, YTHDF2, FTH1, Cardiology, Cell biology, Diseases, Molecular biology

## Abstract

Myocardial ischemia–reperfusion (MI/R) injury remains a major clinical challenge, and ferroptosis has recently emerged as a crucial contributor to its pathogenesis. However, the regulatory mechanisms underlying ferroptosis in MI/R remain incompletely understood. Here, we investigated the role of lactate-mediated YTHDF2 regulation in cardiomyocyte ferroptosis. A murine ischemia–reperfusion (I/R) model and an H9C2 hypoxia/reoxygenation (H/R) model were established. Biochemical assays revealed elevated lactate levels in MI/R hearts, accompanied by increased infarct size, enhanced structural damage, and elevated Fe²⁺ and creatine kinase-MB (CK-MB) levels. Lactate treatment promoted YTHDF2 lactylation and upregulated its expression in cardiomyocytes. Mechanistically, YTHDF2 bound to ferritin heavy chain 1 (FTH1) mRNA and reduced its stability through m6A-dependent degradation, thereby promoting ferroptosis. Knockdown of YTHDF2 suppressed ferroptosis, an effect reversed by FTH1 reduction. These findings identify lactate-induced YTHDF2 lactylation as a key driver of cardiomyocyte ferroptosis and reveal a novel mechanism exacerbating MI/R injury, suggesting that targeting this pathway may represent a potential therapeutic strategy.

**Supplementary Information:**

The online version contains supplementary material available at 10.1038/s41598-026-35130-3.

## Introduction

Ischemic heart disease remains the leading cause of morbidity and mortality worldwide. Although timely coronary reperfusion effectively limits ischemic myocardial injury, myocardial ischemia-reperfusion (MI/R) injury continues to be a major contributor to patient mortality^1^. MI/R injury triggers multiple modes of cell death, including autophagy, apoptosis, pyroptosis, and ferroptosis^2–4^. Notably, prolonged reperfusion markedly enhances ferroptosis, which plays a pivotal role in the chronic cardiac dysfunction associated with MI/R^5^. Therefore, elucidating the molecular mechanisms underlying ferroptosis and identifying novel pharmacological targets are of great importance for developing effective therapeutic strategies against MI/R injury.

In recent years, ferroptosis—a regulated, iron-dependent form of cell death—has gained increasing attention in the context of MI/R injury^6^. Unlike apoptosis or necrosis, ferroptosis is characterized by lipid peroxidation and the accumulation of reactive oxygen species (ROS) due to the inactivation of the lipid repair enzyme glutathione peroxidase 4 (GPX4)^7^. Emerging evidence suggests that ferroptosis contributes significantly to myocardial injury. For example, tanshinone IIA alleviates MI/R injury by inhibiting ferroptosis and apoptosis via voltage-dependent anion channel 1^8^, while circular RNA FEACR suppresses ferroptosis by interacting with nicotinamide phosphoribosyltransferase^9^. Ferritin heavy chain 1 (FTH1) functions as a critical iron-storage protein, oxidizing ferrous (Fe²⁺) to ferric (Fe³⁺) iron and sequestering it in a non-reactive form within the ferritin shell^10^. FTH1 thereby maintains iron homeostasis and protects against ferroptosis. The loss of FTH1 in cardiomyocytes has been shown to enhance ferroptosis and promote cardiomyopathy, whereas protosapanin A exerts cardioprotective effects by facilitating FTH1 autophagic degradation^11^. These findings highlight FTH1 as a key regulator of ferroptosis in the heart^12^. however, the upstream mechanisms controlling its expression during MI/R remain poorly understood.

N6-methyladenosine (m6A) modification plays an essential role in RNA metabolism^13^, affecting mRNA splicing, localization, stability, and degradation through the coordinated actions of methyltransferases (“writers”), demethylases (“erasers”), and m6A-binding proteins (“readers”)^14^. YTH domain family protein 2 (YTHDF2), an m6A reader, selectively binds to m6A-modified transcripts and promotes their decay^15^. Recent studies implicate YTHDF2 in cardiovascular pathologies, where it mediates the degradation of MG53^16^ and SLC7A11^17^ mRNAs, thereby exacerbating MI/R injury and promoting ferroptosis. Bioinformatic analysis suggests that FTH1 mRNA is also a potential YTHDF2 target, implying that YTHDF2 may regulate ferroptosis during MI/R by modulating FTH1 expression.

Ischemia also induces profound metabolic reprogramming in the heart^18^. During reperfusion, oxidative phosphorylation is impaired, forcing cardiomyocytes to rely on glycolysis for energy production^19^. This shift in metabolic strategy leads to a rapid decline in ATP production and an accumulation of lactate, which subsequently causes cellular damage^20^. This shift results in ATP depletion and lactate accumulation, which further aggravates cellular injury. Beyond its metabolic role, lactate can mediate post-translational modifications such as protein lactylation, thereby influencing gene expression^21^. For example, lactate-induced lactylation of Snai1 promotes endothelial–mesenchymal transition and cardiac dysfunction^18^, while lactylation of NLRP3 contributes to inflammatory injury during MI/R^22^. Interestingly, exercise has been shown to mitigate MI/R injury by reducing YTHDF2 lactylation^23^.

These findings suggest that lactate and protein lactylation play critical roles in the regulation of cardiomyocyte fate during MI/R injury. Based on this evidence, we hypothesized that lactate accumulation during MI/R promotes YTHDF2 lactylation, enhancing its ability to mediate m6A-dependent degradation of FTH1 mRNA, thereby triggering ferroptosis and aggravating myocardial injury. Elucidating this lactate–YTHDF2–FTH1 axis may provide novel insights into the mechanisms of MI/R injury and identify potential therapeutic targets for reperfusion therapy.

## Results

### The MI/R mice exhibited elevated lactylation levels

**Fig. 1:**
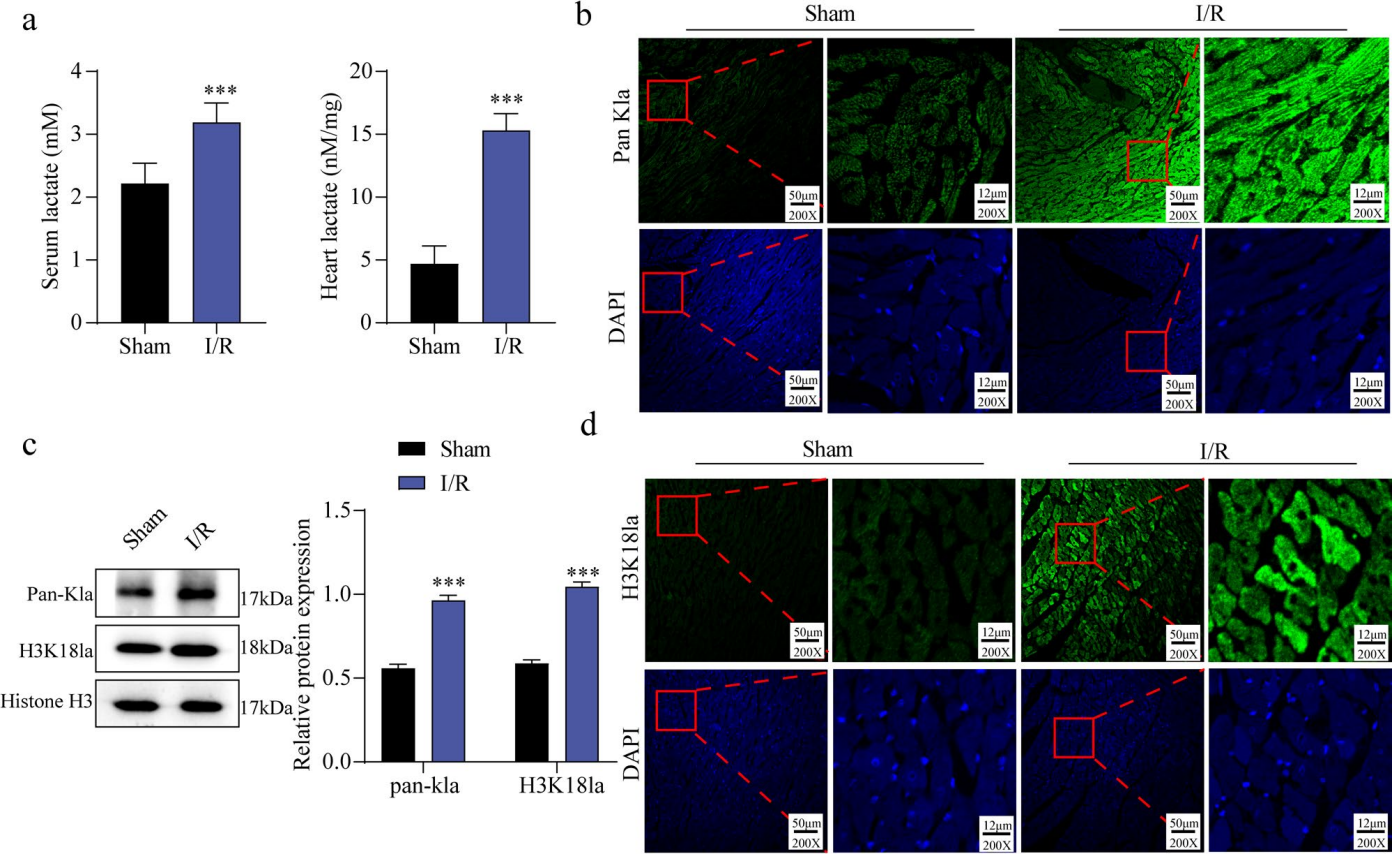
The MI/R mice showed an increase in lactylation levels. C57BL/6 mice are subjected to I/R conditions to establish an MI/R in vivo model, with a sham surgery group as the control group. Serum and myocardial tissue from the mice were subsequently harvested (*n* = 5). (**a**): The colorimetric method was used to detect the lactate levels in mouse myocardial tissue and serum. (**b**): Immunofluorescence staining was employed to confirm Pan Kla expression in myocardial tissue (Scale bar = 50 μm). (**c**): H3K18la and Pan-Kla levels were examined by western blotting. (**d**): Immunofluorescence staining was utilized to validate H3K18la expression in myocardial tissue (Scale bar = 50 μm). Results are from five independent experiments and are expressed as means ± SD. *P < 0.05, **P < 0.01, ***P < 0.001.

To explore MIR injury, a mouse model was established, and changes in lactate levels were assessed. Compared with the Sham group, both serum and myocardial lactate levels were markedly elevated in I/R mice (Fig. 1a). Immunofluorescence staining further revealed that Pan-Kla expression was enhanced in myocardial tissues of I/R mice (Fig. 1b). Western blot analysis also confirmed increased expression of Pan-Kla and H3K18la in the I/R group compared with the Sham group (Fig. 1c). Consistently, immunofluorescence staining demonstrated that H3K18la protein levels were upregulated after I/R induction (Fig. 1d). Collectively, these findings indicate that I/R injury results in elevated lactate levels and enhanced lactylation, as evidenced by increased Pan-Kla and H3K18la expression.

### Reduction of lactate attenuated myocardial injury in I/R mice

**Fig. 2:**
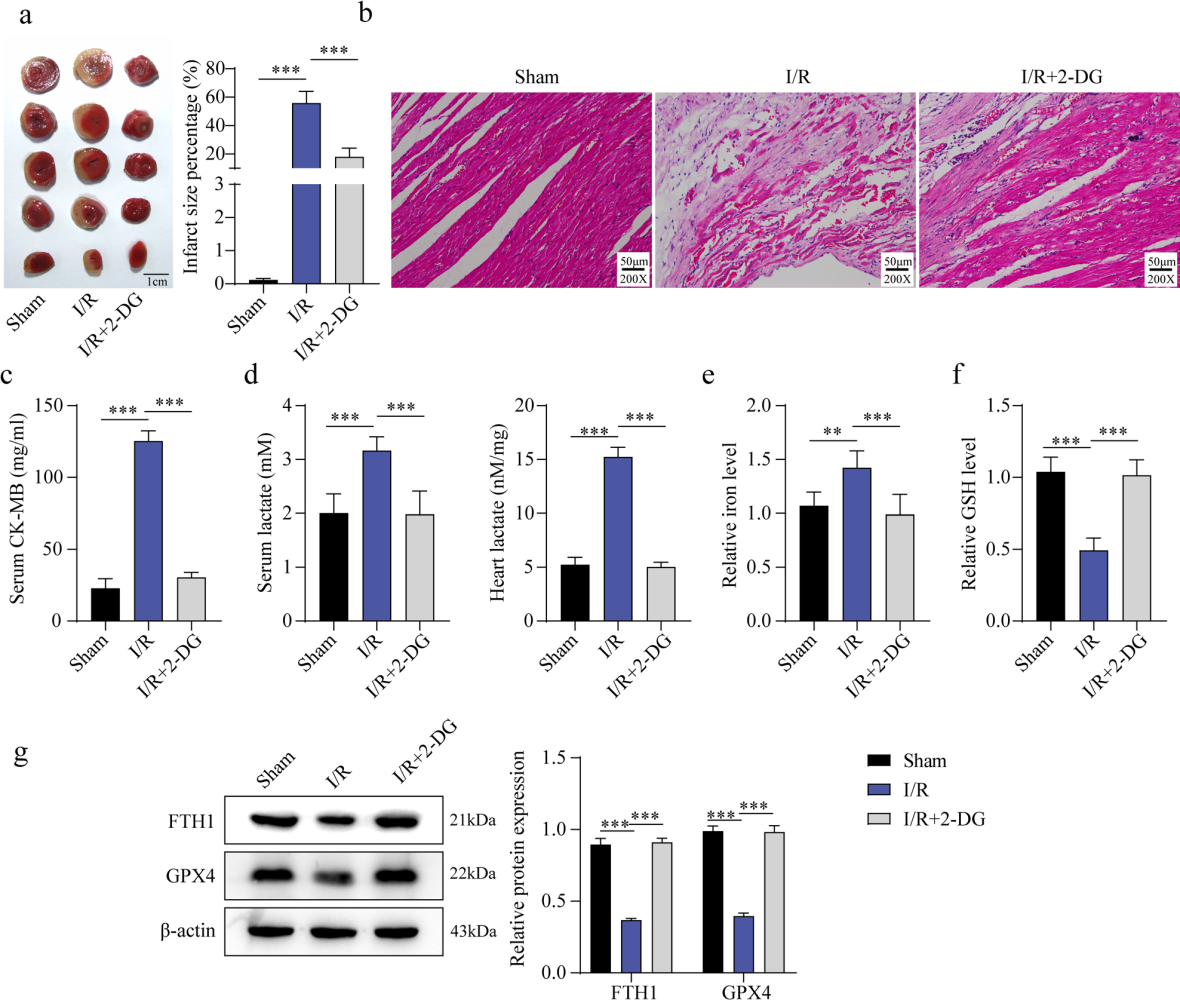
The reduction of lactate alleviated myocardial injury in I/R mice. C57BL/6 mice are exposed to I/R conditions to develop an MI/R in vivo model, using a sham surgery group as the control, and 2-DG was administered (*n* = 5). Subsequently, mice serum and myocardial tissue were collected. (**a**): The area of myocardial infarction was confirmed by TCC staining. (**b**): The pathological condition of myocardial tissue was analyzed through H&E staining (Scale bar = 50 μm). (**c**): The serum CK-MB level of mice was detected by ELISA assay. (**d**): The colorimetric method was used to detect the lactate levels in mouse myocardial tissue and serum. (**e–f**): Commercial kits are utilized to measure Fe^2+^ and GSH levels. (**g**): Western blot was performed to determine the expression of FTH1 and GPX4 in myocardial tissue. Results are from five independent experiments and are expressed as means ± SD. *P < 0.05, **P < 0.01, ***P < 0.001.

To further determine the effect of lactate on myocardial injury, the glycolysis inhibitor 2-deoxyglucose (2-DG) was administered to reduce lactate production. Compared with the Sham group, I/R mice showed a significantly larger infarct area, which was notably reduced following 2-DG treatment (Fig. 2a). Histological analysis revealed that I/R-induced myocardial tissue displayed swelling, degeneration, and necrosis, with diminished transverse striations; these pathological alterations were alleviated by 2-DG (Fig. 2b). Serum CK-MB levels were elevated in I/R mice but decreased after 2-DG administration (Fig. 2c). Similarly, serum and myocardial lactate concentrations were increased by I/R but mitigated by 2-DG treatment (Fig. 2d). Furthermore, Fe²⁺ levels were elevated, and GSH levels were reduced in I/R mice, whereas 2-DG reversed these changes (Fig. 2e–f). Western blot analysis showed that I/R decreased FTH1 and GPX4 expression, both of which were restored following 2-DG treatment (Fig. 2g). In summary, inhibition of lactate production effectively alleviated myocardial injury in I/R mice.

### Lactate promoted YTHDF2 histone lactylation in I/R-Induced cardiomyocytes

**Fig. 3:**
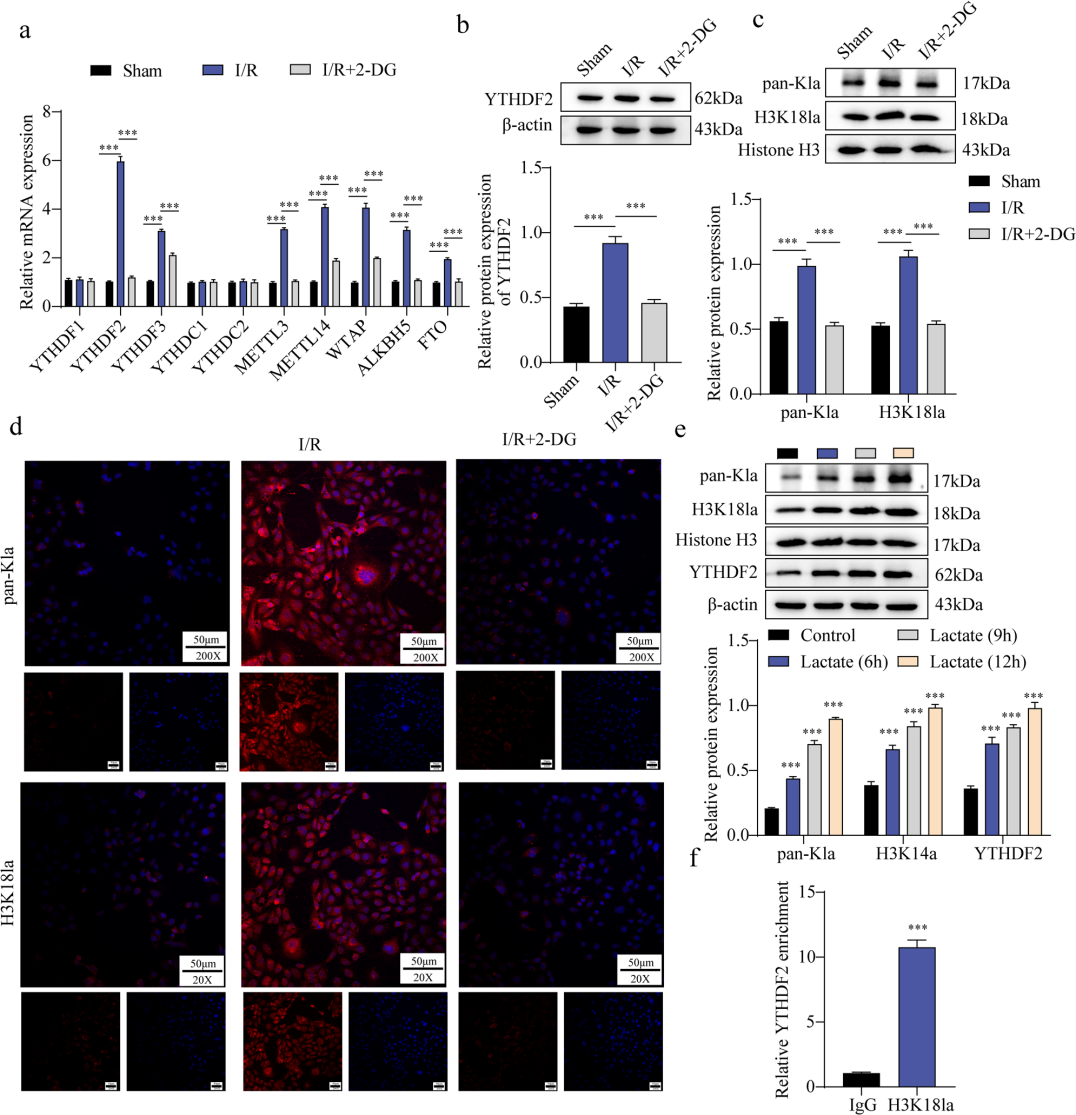
Lactate promoted YTHDF2 histone lactylation in I/R-induced cardiomyocytes. C57BL/6 mice were subjected to I/R and administered with 2-DG. After modeling, myocardial tissue was obtained (*n* = 5). (**a**): The detection of YTHDF1, YTHDF2, YTHDF3, YTHDC1, YTHDC2, METTL3, METTL14, WTAP, AKBH5, and FTO were performed using RT-qPCR. (**b**–**c**): The protein levels of YTHDF2 and pan-Kla were detected by western blot. H9C2 cells were cultured in an H/R environment to mimic the MI/R in vitro model, followed by 2-DG treatment. (**d**): The expression of H3K18la was detected by immunofluorescence staining (Scale bar = 50 μm). H9C2 cells were incubated with 25 mM lactate for different time periods (0, 6, 9, and 12 h). (**e**): The expressions of YTHDF2, H3K18la, and pan-Kla were assessed by western blot. (**f**): The ChIP assay was applied to reveal the interaction between H3K18la and YTHDF2. Results are from five independent experiments and are expressed as means ± SD. *P < 0.05, **P < 0.01, ***P < 0.001.

Given the established role of m⁶A modification in myocardial I/R injury, we examined the expression of m⁶A-related proteins in 2-DG-treated, I/R-induced cardiomyocytes. Among YTHDF2, YTHDF3, METTL14, WTAP, ALKBH5, and FTO, YTHDF2 expression increased most significantly in the I/R group but was inhibited by 2-DG (Fig. 3a). Western blotting confirmed that YTHDF2 protein levels were elevated in I/R mice and suppressed by 2-DG (Fig. 3b). In parallel, Pan-Kla and H3K18la levels were increased in H/R-treated H9C2 cells and reversed by 2-DG (Fig. 3c–d). When cardiomyocytes were exposed to 25 mM lactate for varying durations (0, 6, 9, and 12 h), the expression of YTHDF2, H3K18la, and Pan-Kla progressively increased in a time-dependent manner (Fig. 3e). ChIP analysis further demonstrated enhanced enrichment of H3K18la in the YTHDF2 promoter region compared with the IgG control (Fig. 3f). Collectively, these findings suggest that lactate promotes histone lactylation of YTHDF2 during H/R-induced injury.

### YTHDF2 knockdown upregulated FTH1 and suppressed H/R-induced ferroptosis in H9C2 cells

**Fig. 4:**
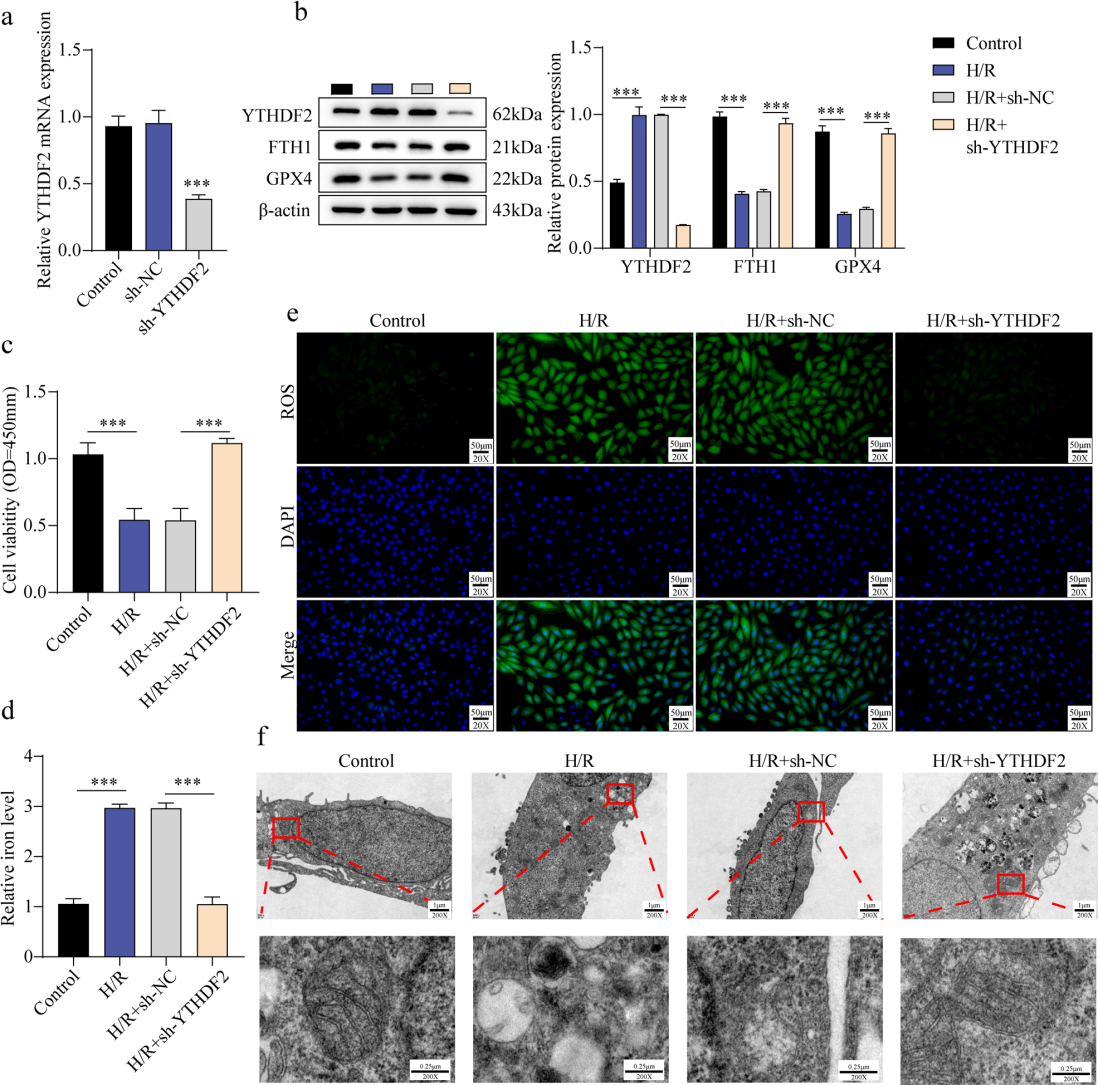
Knockdown of YTHDF2 upregulated FTH1 to inhibit H/R-stimulated ferroptosis in H9C2 cells. H9C2 cells underwent transfection with sh-YTHDF2 lentivirus and the corresponding control and induced by H/R conditions. (**a**): RT-qPCR used to check the mRNA level of YTHDF2. (**b**): Using western blot, the levels of YTHDF2, GPX4, and FTH1 were determined. (**c**): The method of CCK-8 was employed to detect cell viability. (**d**): Commercial kits were used to detect Fe^2+^ concentration. (**e**): The level of ROS was checked by immunofluorescence staining (Scale bar = 50 μm). (**f**): The mitochondrial morphology was observed by TEM (Scale bar = 1 μm). Results are expressed as means ± SD. *P < 0.05, **P < 0.01, ***P < 0.001.

To elucidate the function of YTHDF2 in H/R-stimulated cardiomyocytes, YTHDF2 was silenced in H9C2 cells prior to H/R treatment. H/R exposure significantly increased YTHDF2 mRNA and protein expression, whereas YTHDF2 knockdown abrogated this upregulation (Fig. 4a–b). Conversely, GPX4 and FTH1 expression were reduced following H/R treatment, and YTHDF2 inhibition reversed these decreases (Fig. 4b). H/R exposure reduced cell viability, which was restored upon YTHDF2 knockdown (Fig. 4c). Moreover, H/R-induced elevations in Fe²⁺ and ROS levels were attenuated by YTHDF2 silencing (Fig. 4d–e). TEM analysis revealed that mitochondria in the H/R group appeared smaller with disrupted cristae and condensed membranes, whereas YTHDF2 knockdown ameliorated these morphological changes (Fig. 4f). Together, these findings indicate that suppression of YTHDF2 enhances FTH1 expression and mitigates ferroptosis in H/R-induced cardiomyocytes.

### YTHDF2 facilitated FTH1 mRNA degradation

**Fig. 5:**
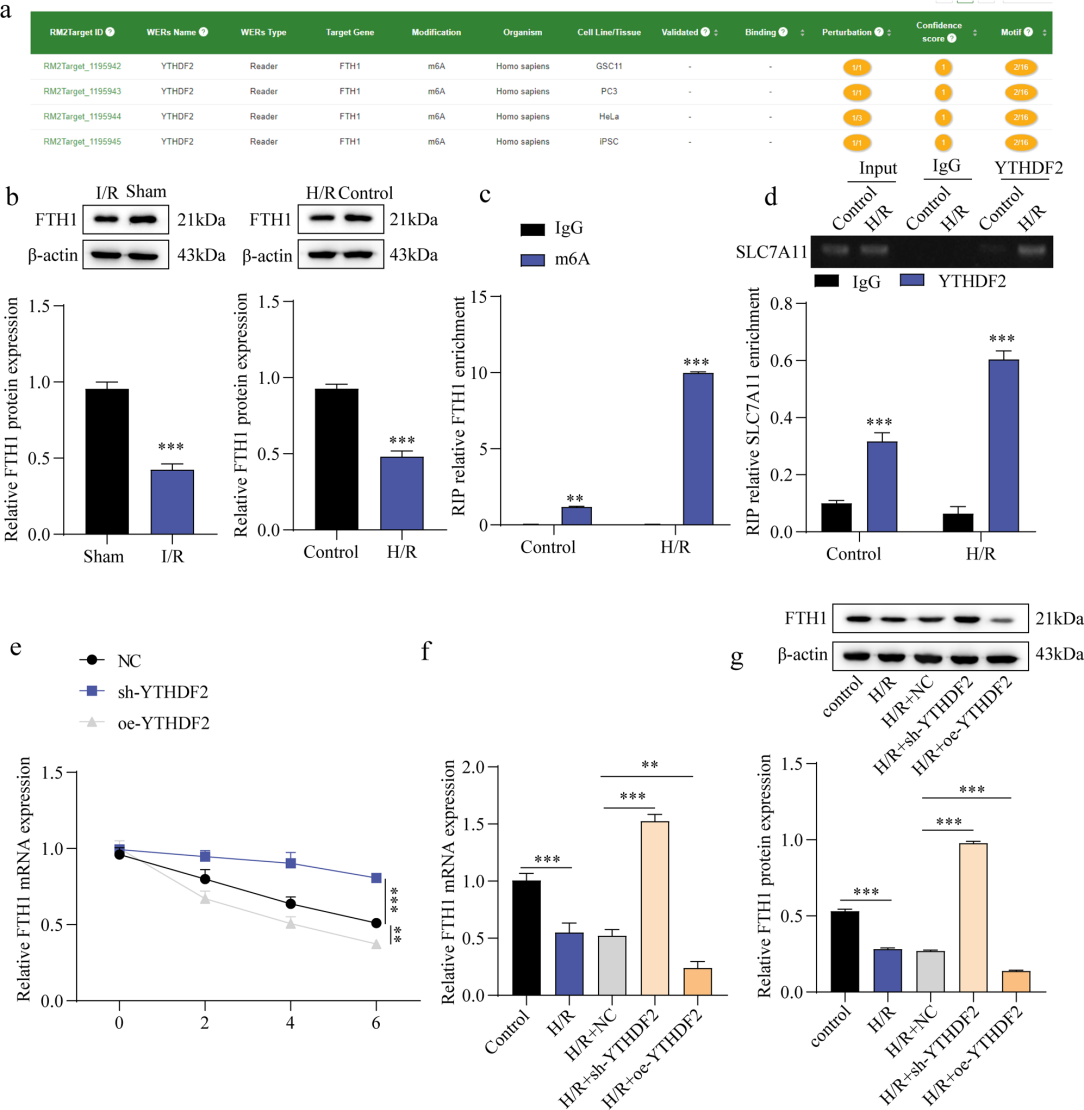
YTHDF2 accelerated the degradation of FTH1 mRNA. (**a**): Prediction of m6A modification of FTH1 on the SRAMP website. (**b**) The expression of FTH1 in both the I/R mice model (*n* = 5) and the H/R H9C2 cell model. was identified through western blotting. (**c**): The enrichment of m6A on FTH1 mRNA was analyzed by MeRIP-qPCR. (**d**): The RIP assay was conducted to verify the link between FTH1 and YTHDF2. (**e**): Constructing YTHDF2 overexpression or knockdown H9C2 cells and administering with actinomycin D. (**f**–**g**): The detection of FTH1 levels was carried out through RT-qPCR and western blot. Results are expressed as means ± SD. *P < 0.05, **P < 0.01, ***P < 0.001.

Prediction from the SRAMP database revealed potential m⁶A modification sites within FTH1 mRNA (Fig. 5a). Both in vivo and in vitro models of MIRI showed reduced FTH1 expression (Fig. 5b). The m⁶A enrichment of FTH1 mRNA was significantly elevated in the H/R group (Fig. 5c). RIP analysis confirmed that YTHDF2 directly binds to FTH1 mRNA, and this interaction was enhanced under H/R conditions (Fig. 5d). Furthermore, YTHDF2 inhibition slowed FTH1 mRNA decay, whereas its overexpression accelerated degradation (Fig. 5e). Consistently, H/R-induced suppression of FTH1 expression was reversed by YTHDF2 knockdown but further reduced by YTHDF2 overexpression (Fig. 5f–g). Collectively, these results demonstrate that YTHDF2 negatively regulates FTH1 mRNA stability by promoting its degradation.

### FTH1 knockdown reversed the protective effect of YTHDF2 silencing on cardiomyocyte ferroptosis

**Fig. 6:**
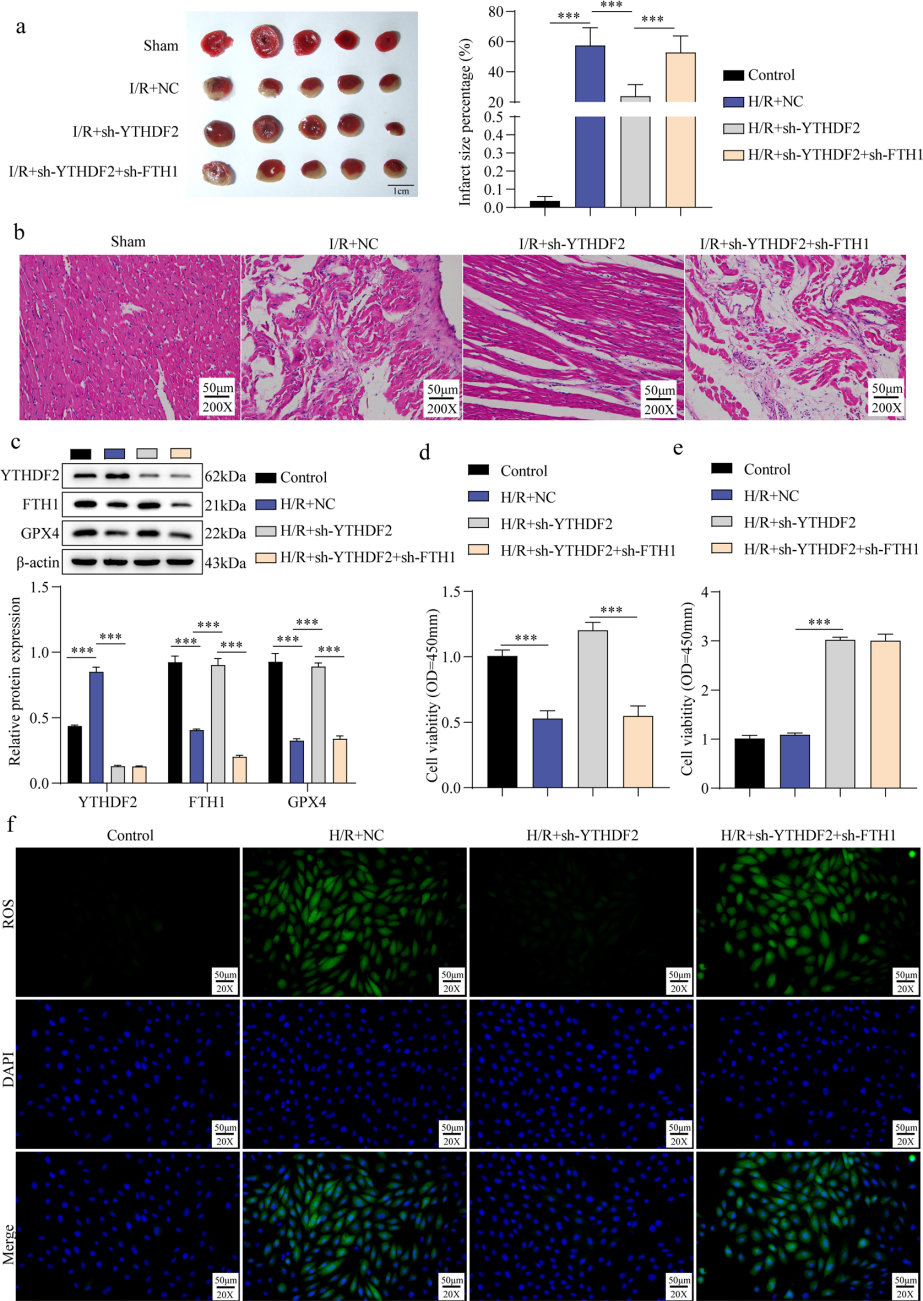
Knockdown of FTH1 alleviates the inhibition of YTHDF2 downregulation on cardiomyocyte ferroptosis. Knocking down YTHDF2 and/or FTH1 in C57BL/6 mice, followed by I/R induction (*n* = 5). (**a**): The myocardial infarction area was verified using TCC staining. (**b**): H&E staining was employed to analyze the pathological condition (Scale bar = 50 μm). Knocking down YTHDF2 and/or FTH1 in H9C2 cells, followed by H/R induction. (**c**): Western blot was used to detect the levels of YTHDF2 and FTH1. (**d**): Cell activity was measured using CCK-8 assay. (**e**): The measurement of Fe^2+^ levels is done with commercial kits. (**f**): Identifying ROS levels by using immunofluorescence staining (Scale bar = 50 μm). Results are expressed as means ± SD. *P < 0.05, **P < 0.01, ***P < 0.001.

To investigate the regulatory relationship between YTHDF2 and FTH1 in cardiomyocyte ferroptosis, YTHDF2 and/or FTH1 knockdown mice were generated and subjected to I/R injury. Myocardial infarct size was markedly increased in the I/R group compared to the Sham group. YTHDF2 knockdown significantly reduced infarct size, whereas simultaneous FTH1 silencing reversed this effect (Fig. 6a). H&E staining revealed that I/R-induced myocardial tissue exhibited swelling, necrosis, and disrupted transverse striations, which were ameliorated by YTHDF2 knockdown but restored by FTH1 inhibition (Fig. 6b). In H/R-treated H9C2 cells, YTHDF2 was upregulated while FTH1 and GPX4 were downregulated. YTHDF2 silencing decreased YTHDF2 expression while increasing FTH1 and GPX4, whereas FTH1 knockdown suppressed FTH1 and GPX4 without affecting YTHDF2 (Fig. 6c). The enhanced cell viability observed after YTHDF2 knockdown was abolished by FTH1 silencing (Fig. 6d). Similarly, reductions in Fe²⁺ and ROS levels caused by YTHDF2 knockdown were reversed when FTH1 was silenced (Fig. 6e–f). In conclusion, YTHDF2 promotes cardiomyocyte ferroptosis by facilitating FTH1 downregulation.

## Discussion

MI/R injury accounts for nearly half of all cardiac injury cases and can lead to severe clinical outcomes, including arrhythmia, heart failure, and even cardiac arrest^24^. To better prevent MI/R injury, it is essential to clarify its underlying molecular mechanisms. In this study, we explored how lactate induces ferroptosis in both an I/R mouse model and H9C2 cells subjected to hypoxia/reoxygenation (H/R). We observed a marked elevation of lactate production during MI/R, accompanied by enhanced protein lactylation and upregulation of YTHDF2 expression. Importantly, lactate promoted the lactylation of YTHDF2, which in turn recognized the m6A site on FTH1 mRNA, promoting its degradation. This molecular cascade triggered ferroptosis and aggravated myocardial injury during MI/R.

Epigenetic regulation, including histone lactylation, plays a critical role in maintaining cellular homeostasis and in driving various physiological and pathological processes^25^. Lactylation not only regulates cell proliferation, differentiation, epithelial-mesenchymal transition (EMT), inflammatory responses, and tissue fibrosis, but also influences ferroptosis—a key determinant in the progression of several diseases^26,27^. The heart, with its exceptionally high energy demand, relies predominantly on fatty acid β-oxidation for ATP production, while glucose oxidation and glycolysis provide supplementary energy sources^20,28^. Under ischemic conditions, the myocardium shifts toward anaerobic glycolysis to adapt to hypoxia, resulting in excessive lactate accumulation^18^. Previous studies have demonstrated that lactate acts as a metabolic signaling molecule that can modulate cardiac injury. For example, HSPA12A maintains glycolytic balance and histone H3 lactylation to mitigate MI/R injury^29^. Consistent with these findings, our study revealed significantly elevated lactate levels and lactylation modifications in MI/R mouse hearts and H/R-treated H9C2 cells. Furthermore, treatment with 2-DG, a glycolysis inhibitor, markedly reduced lactylation levels, supporting the notion that glycolysis-derived lactate drives this modification. However, following specific inhibition of glycolysis by 2-DG, MI/R can maintain intracellular energy homeostasis through three core metabolic pathways. Concurrently, the reversal of mitochondrial permeability transition (MPT) contributes to the partial recovery of oxidative phosphorylation (OXPHOS) function. Specifically, the rapid activation of the phosphagen system provides immediate energy buffering for cardiomyocytes in the early phase of reperfusion^30^. Compensatory upregulation of fatty acid oxidation then serves as the primary source of medium- to long-term energy supply^20^. Additionally, the induction of ketone body metabolism acts as an auxiliary energy pathway, supplementing ATP production^31^. The reversal of MPT, by preserving mitochondrial structure and functional integrity, provides a crucial subcellular foundation for the efficient operation of the aforementioned metabolic pathways. Together, these mechanisms coordinately restore the balance between energy supply and demand under MI/R stress^32^.

Ferroptosis has been widely recognized as a key form of cell death during MI/R injury^33^. It is characterized by excessive iron accumulation, ROS overproduction, and subsequent lipid peroxidation^34^. Within this pathway, FTH1 serves as a crucial regulator by sequestering free iron and limiting ferroptotic damage^35^. Our findings uncovered a novel interaction between YTHDF2 and FTH1: YTHDF2 binds to the m6A-modified region of FTH1 mRNA and destabilizes it, thereby promoting ferroptosis in cardiomyocytes under MI/R conditions. This is in line with previous studies showing that YTHDF2 modulates m6A-dependent degradation of transcripts such as BNIP3 and SLC7A11 to influence MI/R outcomes^17,36^. Moreover, YTHDF2 undergoes lactylation modification during MI/R, which further accelerates the pathological process^23^. Our study extends these findings by demonstrating that lactylation of YTHDF2 specifically promotes ferroptosis, rather than other forms of cell death, thereby establishing a new regulatory axis in MI/R pathology. This represents a key novelty of our work. Different from previous report^23,37^, while lactate-mediated YTHDF2 lactylation and YTHDF2-dependent FTH1 degradation have been documented separately, our research is the first to demonstrate that lactylation modification directly targets and modulates YTHDF2’s regulatory function on FTH1 in the context of cardiomyocyte ferroptosis during MI/R. This finding establishes a direct mechanistic link between lactate metabolism, post-translational modification of YTHDF2, and iron homeostasis, expanding the understanding of the “lactate-YTHDF2-FTH1 axis” beyond isolated molecular events to a functional, context-specific regulatory cascade in myocardial injury.

Research indicated that a reduction in pH induces lysosomal iron release^38^, which, in conjunction with YTHDF2-mediated downregulation of FTH1, may significantly elevate the intracellular labile iron pool, thereby providing an increased supply of “iron substrates” conducive to ferroptosis. In studies on glioblastoma, it has been observed that the EGFR/SRC/ERK signaling pathway can stabilize the YTHDF2 protein and enhance its mRNA-binding activity through phosphorylation at the S39 and T381 sites of YTHDF2. Phosphorylated YTHDF2 exhibits an enhanced ability to recognize and bind m6A-modified mRNA, thereby promoting mRNA degradation or altering its translation efficiency. The MEK/ERK pathway, mediated by GRP81, is the mechanism through which lactate influences skeletal muscle^39^. Consequently, upon activation of GPR81, YTHDF2 may undergo phosphorylation via the ERK signaling pathway, thereby augmenting its efficiency in degrading FTH1 mRNA and creating a “synergistic amplification” effect through the direct interaction of YTHDF2 with lactate.

Despite these findings, several limitations should be acknowledged. First, the relatively small sample sizes of both the I/R mouse and H/R cell models may limit the robustness and generalizability of the conclusions. Future studies with larger cohorts are warranted. Second, in our in vitro experiments, we exclusively utilized H9C2 rat embryonic cardiomyocytes. This limitation in our study design, namely the absence of validation using primary cardiomyocytes or human induced pluripotent stem cell-derived cardiomyocytes (iPSC CMs), constrains the direct applicability of our conclusions to primary or human cardiomyocytes. Future research endeavors will incorporate primary cardiomyocytes and iPSC CMs to assess the conservation of the identified mechanisms across different cell models and species, thereby enhancing the generalizability of our findings. Third, our findings lack validation using clinical samples. Given the physiological and metabolic differences between murine and human hearts, as well as the presence of comorbidities such as hypertension, diabetes, and hyperlipidemia in patients with myocardial infarction, translating these results to clinical contexts requires careful consideration. Finally, while we identified the involvement of YTHDF2 lactylation in ferroptosis, further investigation is needed to pinpoint the precise lactylation sites and delineate their functional roles in MI/R injury.

In conclusion, this study reveals a novel mechanism in which lactate acts as a signaling molecule to induce YTHDF2 lactylation during MI/R injury. This modification promotes m6A-dependent degradation of FTH1 mRNA, triggering ferroptosis and exacerbating myocardial damage. These findings deepen our understanding of the molecular mechanisms underlying reperfusion injury and provide new insights for developing targeted therapeutic strategies against MI/R-induced cardiac injury.

## Materials and methods

### Establishment of MI/R injury mouse model

Male C57BL/6 mice (8 weeks old, 20–22 g) were purchased from SJA (Hunan, China). Animals were housed at 22 °C under a 12 h light/dark cycle with free access to standard chow and water. Mice were anesthetized with 2% isoflurane (MedChemExpress, USA) and positioned supine on a 37 °C heating pad for left thoracotomy under mechanical ventilation. Myocardial I/R injury was induced by occluding the left anterior descending coronary artery (LAD) for 45 min, followed by 24 h of reperfusion. Sham-operated mice underwent the same surgical procedures without LAD occlusion. Lentiviral vectors (LV-sh-NC, LV-sh-YTHDF2, and LV-sh-FTH1) were obtained from GenePharma (Shanghai, China) and administered to the experimental animals. According to published research^40^, the lentiviral vectors were administered before the surgical procedure (not at the time of reperfusion) and were delivered via local intramyocardial injection. Additionally, 2-deoxyglucose (2-DG; Sigma-Aldrich, USA) was administered intraperitoneally at 100 mg/kg one hour before model induction. As for euthanasia procedure, At the end of the 24 h reperfusion period, all experimental mice (including sham-operated mice) were euthanized using a chemically induced method compliant with the American Veterinary Medical Association (AVMA) Guidelines for the Euthanasia of Animals. First, mice were anesthetized with 5% isoflurane (MedChemExpress, USA) via a rodent anesthesia induction chamber (Model: R500, RWD Life Science, China) to ensure loss of consciousness (verified by absence of pedal withdrawal reflex). Then, euthanasia was completed by intraperitoneal injection of sodium pentobarbital (Sigma-Aldrich, USA) at a dose of 150 mg/kg body weight. Death was confirmed by the absence of respiratory movement, cardiac pulse (palpated via the chest wall), and corneal reflex for at least 5 min. All animal experiments were approved by the Institutional Ethics Committee of Xiangya Hospital. and conducted in accordance with the standards of the International Association for the Study of Pain (IASP). This study is also reported in accordance with ARRIVE guidelines (https://arriveguidelines.org).

### Cell culture and treatment

Rat cardiomyoblast H9C2 cells (Pricella, Wuhan, China) were maintained in Dulbecco’s modified Eagle’s medium (DMEM; Gibco, USA) supplemented with 10% fetal bovine serum (Pricella) and 1% penicillin–streptomycin (Coolaber, Beijing, China) at 37 °C in a humidified atmosphere containing 5% CO₂. The culture medium was replaced every two days, and cells were subcultured when 60% confluent. For treatment, cells were exposed to 5 mM lactate (LA; Sigma-Aldrich) or 10 mM 2-DG (Sigma-Aldrich) for 1 h to assess the role of lactylation in MI-R injury^41^.

### Plasmids, transfection, lentivirus production and infection

Lentiviruses expressing YTHDF2 (oe-YTHDF2) or control vector (oe-NC) were obtained from GenePharma. H9C2 cells were infected at a multiplicity of infection (MOI) of 10 in the presence of 5 µg/mL Polybrene (GenePharma). After transduction, cells were selected with 5 µg/mL puromycin (Sigma-Aldrich) for subsequent experiments. For gene knockdown, shRNAs targeting YTHDF2 (5′-GATGGATTAAACGATGATGAT-3′) and FTH1 (5′-GCCTCGGGCTAATTTCCCATA-3′) were synthesized by GenePharma, with a non-targeting shRNA (sh-NC) as control. Transfections were performed using Lipofectamine™ 3000 (Thermo Fisher Scientific, USA).

### Establishment of the hypoxia/reoxygenation (H/R) cell model

To mimic MI/R injury in vitro, H9C2 cells were incubated in a hypoxic chamber (95% N₂, 5% CO₂, 37 °C) for 2 h, followed by reoxygenation for 4 h in an atmosphere of 75% N₂, 20% O₂, and 5% CO₂. Control cells were maintained under normoxic conditions (95% air, 5% CO₂) for 6 h.

### Immunofluorescence staining

After treatment, cells on coverslips were fixed with 4% paraformaldehyde, permeabilized with 0.1% Triton X-100 for 10 min, and blocked with 5% bovine serum albumin (BSA) for 30 min. Cells were then incubated with primary antibodies overnight at 4 °C, followed by fluorescent secondary antibodies for 1 h at room temperature in the dark. Nuclei were counterstained with DAPI for 10 min, and images were captured using an Olympus fluorescence microscope (Tokyo, Japan).

### Cell counting Kit-8 (CCK-8) assay

H9C2 cells were seeded in 96-well plates (8 × 10³ cells/well) and transfected as indicated. Cell viability was measured using a Cell Counting Kit-8 (CCK-8; Sigma-Aldrich). After adding 10 µL of CCK-8 reagent per well, cells were incubated for 3.5 h at 37 °C, and absorbance was measured at 450 nm using a microplate reader (Thermo Fisher Scientific).

### Lipid ROS measurement

Lipid ROS levels were determined using BODIPY™ 581/591 C11 (Thermo Fisher Scientific). Cells were washed twice with pre-warmed PBS and incubated with 2.5 µM dye for 30 min at 37 °C in the dark. After washing with PBS, fluorescence was imaged using an Olympus microscope, and intensity was quantified with ImageJ (NIH, USA).

### Hematoxylin–eosin (H&E) staining

The heart tissue from the mice, which was damaged by reperfusion, was preserved in paraffin, sliced into section 5 μm thick, and stained with an improved H&E staining kit (Sorabio, Beijing, China). The slices were sealed with neutral resin, and then we used an inverted microscope (Nikon Ti2, Tokyo, Japan) to take pictures.

### The 2,3,5-Triphenyl tetrazolium chloride (TTC) staining

Seven days following the establishment of the I/R model, mice were euthanized under anesthesia for sample collection. TTC staining (Solarbio) was employed to assess the extent of necrotic myocardium. The hearts were exercised and preserved at − 80 °C. Subsequently, the hearts were sectioned into 1-mm-thick transverse slices and stained with 2% TTC for 15 min at 37 °C. The necrotic regions, which appeared white, were imaged and quantified using ImageJ software (National Institutes of Health).

### Lactate measurement

Blood samples were taken from each group of mice and centrifuged at 3000 rpm for 15 min, after which lactate levels were measured with a colorimetric assay as instructed by the manufacturer (Solarbio).

### Enzyme-linked immunosorbent assay (ELISA)

Two hours post-reperfusion, blood samples were taken from the heart cavity of the mice. Following centrifugation, serum was extracted, and a biochemical analyzer (Siemens, Munich, Germany). was used to determine the CK–MB concentrations in the serum samples from each group of mice.

### GSH and Fe^2+^ detections

GSH levels were measured using a GSH assay kit (Solarbio) according to the manufacturer’s instructions. Optical density was read at 412 nm. Intracellular Fe²⁺ content was determined using a ferrous iron assay kit (Solarbio) and the fluorescent probe FeRhoNox-1 (MKBio, Shanghai, China).

#### Methylated RNA binding protein Immunoprecipitation (Me-RIP)

Total RNA was extracted with TRIzol reagent (Thermo Fisher Scientific). Samples were incubated with m6A antibody (1:500, ab151230, Abcam) or IgG control (1:100, ab109489, Abcam) and protein A/G magnetic beads. After overnight incubation with IP buffer, RNA was eluted, purified, and analyzed by real-time quantitative polymerase chain reaction (RT-qPCR) to determine m6A modification of FTH1 mRNA.

### Western blot

Proteins were quantified post-separation using a kit for bicinchoninic acid assay procured from Beyotime (Shanghai, China). A total of 20 µg of protein was resolved on a 10% SDS-PAGE gel and subsequently transferred onto PVDF membranes (Millipore, MA, USA). The membranes were blocked with 5% non-fat milk in TBST for one hour and then incubated with specific primary antibodies at 4 °C overnight. After being washed three times with TBST, the membranes were exposed to secondary antibodies linked to horseradish peroxidase and then incubated overnight with antibodies specific to H3K18la (1:500; PTM-1406RM, PTM BIO), Pan-Kla (1:1000, PTM-1401RM, PTM BIO), FTH1 (1:1000, ab75973, Abcam), GPX4 (1:1000, ab125066, Abcam), YTHDF2 (1:1000, ab220163, Abcam), Histone H3 (1:5000; PTM-1002RM, PTM BIO), and β-actin (1 µg/ml, ab8226, Abcam). Detection of protein bands was performed using enhanced chemiluminescence (ECL) from Beyotime, and ImageJ software was employed to analyze the grayscale intensity of the bands. Antibody dilutions were prepared following the manufacturer’s guidelines.

### RT-qPCR

Total RNA was extracted using TRIzol, and 1 µg RNA was reverse transcribed into cDNA using the PrimeScript™ RT kit (Takara, Japan). qPCR was performed using SYBR Green Master Mix (Roche, Switzerland) on a Thermo Fisher instrument. GAPDH served as an internal control, and relative expression was calculated by the 2^−ΔΔCt method. Primer sequences are listed in Table 1.

**Table 1:** The primers for RT-qPCR.

Gene	Forward primer (5′-3′)	Reverse primer (5′-3′)
YTHDF1	ATGTCGGCCACCAGCGTGGACA	TCATTGTTTGTTTCGACTCTGC
YTHDF2	TGTTGGAGAAGCTTCGGTCC	ACCCGGCCATGTTTCAGATT
YTHDF3	GGTGTATTTAGTCAACCTGGGG	AAGAGAACTAGGTGGATAGCCAT
YTHDC1	AACTGGTTTCTAAGCCACTGAGC	GGAGGCACTACTTGATAGACGA
YTHDC2	CAAAACATGCTGTTAGGAGCCT	CCACTTGTCTTGCTCATTTCCC
METTL3	AACTGCAACGCATCATTCGG	TGACTGGTGGAACGAACCAA
METTL14	GAGCTGAGAGTGCGGATAGC	GCAGATGTATCATAGGAAGCCC
WTAP	AGTGCACCACTCAAATCCAGT	AGGCGTAAACTTCCAGGCA
ALKBH5	CGGCGAAGGCTACACTTACG	CCACCAGCTTTTGGATCACCA
FTO	GCTGCTTATTTCGGGACCTG	AGCCTGGATTACCAATGAGGA
FTH1	ACATCAAGAAGGTGGTGAAGC	AAGGTGGAAGAGTGGGAGTTG
GAPDH	TCTTAAGAAGACGACGGCTTCAG	TTGCTCTCTCACTTGTCCTCGAT

### Chromatin immunoprecipitation (ChIP)

After treatment, cells were cross-linked with paraformaldehyde, quenched with glycine, lysed, and sonicated. Chromatin was immunoprecipitated using antibodies against IgG, H3K18la, or YTHDF2 with protein A/G beads. Complexes were washed, eluted, and reverse cross-linked. Purified DNA fragments were analyzed by RT-qPCR (Table 2).

**Table 2:** The primers for ChIP.

Gene	Forward primer (5′-3′)	Reverse primer (5′-3′)
YTHDF2	TTGTACCTGGACTCGGGAGA	CCAGTCAACCTCATGGCTCA

### RNA immunoprecipitation (RIP)

The RIP analysis was performed to confirm the interactions between YTHDF2 and FTH1, using the RIP Kit (Sigma-Aldrich). Following the lysis of H9C2 cells in RIP lysis buffer, 15 µg of the extracted cell protein was added to protein A/G magnetic beads with anti-IgG (#ab172730, 4 µg, Abcam) and anti-YTHDF2 (#ab220163, 4 µg, Abcam) antibody, then kept at 4 °C for an hour. Following this, protease K was used to isolate the co-precipitated RNA, and qRT-PCR and immunoblotting analysis were conducted to evaluate the relative expression of FTH1.

### RNA decay assay

H9C2 cells were respectively transfected with NC, sh-YTHDF2 and oe-YTHDF2 lentivirus vectors, separately, followed by stimulation with Actinomycin D (5 µg/mL, A606804, Sangon Biotech, Shanghai) for 2, 4, and 6 h. Subsequently, H9C2 cells were collected for the measurement of FTH1 mRNA levels via RT-qPCR analysis.

### Transmission electron microscope (TEM)

Cells were treated with 2.5% glutaraldehyde and 1% osmium for fixation and then dehydrated using ethanol at different concentrations. For 2 h each, the cells were treated with a 1:1 combination of epoxy propane and epoxy resin, as well as with pure epoxy resin. They were then coated with pure epoxy resin and heated at 40 °C for 12 h, followed by 60 °C for 48 h. Finally, the cells were stained with lead and uranium salts and observed by a TEM (Hitachi, Tokyo, Japan).

### Statistical analysis

The data were collected from at least three separate trials, and the statistical analysis, presented as mean ± SD, was conducted using GraphPad Prism 9.0 software. Student’s t-test was employed to assess differences between two groups, whereas one-way ANOVA with Tukey’s test was used for comparing multiple groups. A *P*-value of less than 0.05 was considered statistically significant.

## Supplementary Information

Below is the link to the electronic supplementary material.

Supplementary Material 1

## Data Availability

The data sets generated or analyzed during this study are available from the corresponding author upon reasonable request.
